# Development of a Novel Flavored Goat Cheese with *Gentiana lutea* Rhizomes

**DOI:** 10.3390/foods12030468

**Published:** 2023-01-19

**Authors:** Christian Coelho, Cécile Bord, Karine Fayolle, Cindy Bibang, Stéphanie Flahaut

**Affiliations:** 1 INRAE, VetAgro Sup Campus Agronomique de Lempdes, UMR F, Université Clermont Auvergne, 15000 Aurillac, France; 2 CPPARM, ZA Les Quintrands, Route de Volx, 04100 Manosque, France

**Keywords:** flavored cheese, *Gentiana lutea* rhizomes, volatile, bitterness, olfaction

## Abstract

*Gentiana lutea* rhizomes, generally used as a bittering agent in food, were harvested from two geographical sites (Massif Central: MC and Jura: J) to evaluate their potential use in the flavoring step during goat cheesemaking. Gentian flavored goat cheeses (MCGC and JGC) were elaborated by a one-night immersion of unflavored goat cheeses (CGC) into gentian-infused whey. The impregnation of gentian in goat cheeses was evaluated by chemical and sensory analysis. The chemical composition of cheeses was analyzed by HS-SPME-GC-MS (Head-Space—Solid Phase MicroExtraction—Gas Chromatography—Mass Spectrometry) for volatile compounds (alcohols, ketones, aldehydes, esters, alkenes, alkanes, acids, terpenes) and UHPLC-DAD (Ultra High-Performance Liquid Chromatography—Diode Array Detector) for gentian bitter compounds (seco-iridoids). The sensory analysis consisted of a bitterness rating and a free description of cheeses by 17 trained panelists. Results of the study highlighted that unflavored goat cheeses presented higher unpleasant notes (goaty and lactic whey) and higher amounts of hexanoic acid and toluene compared to gentian flavored goat cheeses. The bitterness of gentian flavored goat cheeses was higher compared to unflavored cheeses and could be explained by loganic acid transfer from yellow gentian to flavored cheeses. Other free descriptors of gentian flavored goat cheeses revealed more complex notes (herbal, vegetal, floral, sweet, spicy and creamy) and higher relative amounts of volatile compounds such as 3-methyl butanoic acid, 2-methyl propanoic acid, 4-methyl decane, 2,3-butanediol, ethanol, diacetyl, methyl acetate and 2-phenylethyl acetate, compared to unflavored cheeses. Phenylethyl acetate was the only volatile compound that enabled differentiation of gentian origin on gentian flavored goat cheeses. Gentian rhizomes could be considered a promising flavoring agent contributing to the olfactive and gustative complexity of flavored goat cheeses and the reduction of their goaty perceptions.

## 1. Introduction

Cheese represents the food product obtained exclusively with milk-producing materials: (milk, partly or wholly skimmed milk, cream, fat, buttermilk) that has coagulated with partial removal of the aqueous part. Most of the time, fermentation and ripening stages are achieved during cheese making, particularly on most of PDO (Protected Denomination of Origin) and PGI (Protected Geographical Indication) cheeses. During cheesemaking, the use of additives is regulated (EU 2007-628) and limited to a maximum of 30 % of the final cheese product. Some of these additives are completely part of the production specifications. This is the case with ingredients such as rennet, fermentative microorganisms, and salt. Salt is mostly used in order to avoid microbiological spoilage and to increase cheese flavor quality [1]. Rennet and microorganisms contain and/or liberate clotting enzymes such as chymosin or pepsin and other lipases/proteases that hydrolyze the casein chains in milk and permit their coagulations [2,3]. Their use during cheese making, particularly microorganisms, leads to metabolic changes (consumption of carbon and nitrogen substrates, curd acidification, decrease in redox potential, lipolysis, proteolysis) in the dairy matrix, conferring to the neo formed cheese elevated amounts of biochemical metabolites contributing to tasty and odorous characteristics [4,5]. Numerous studies illustrated the use of specific strains during cheese making for their role in mouthfeel perceptions and aromatization [6,7,8,9]. Recent studies also highlighted that the presence of native microbial communities in raw milk and their evolution during cheesemaking steps could directly affect the pathways associated with volatile metabolite production in cheeses [10,11].

Nevertheless, non-conventional flavoring processes received a boost these last years in dairy products, with the objectives to diversify the taste and to propose multi-functional products with increased nutritional and health properties for consumers. Among them, physical thermal processes can be applied to confer a smoky and toasty character to cheese by direct infusion of burning wood vapors in contact with cheese [12,13]. Other strategies evaluated the direct incorporation of phenolic liquids smokes to cheese [13] or the use of uncommon vegetable material for burning purposes [14]. Other flavoring techniques consisted of directly using plant-based proteins originating from vegetal milk to propose cheese substitutes [15]. Many other studies focused on the use of herbal plants blended with the curds to offer a diversity of aromatized cheeses products [4,16,17,18,19,20,21,22,23]. Their aromatic characteristics are generally enhanced in terms of spicy, fruity, and floral character. However, its consumer acceptability could be controversial [21,24] due to flavor perception modifications occurring among cheese tasty compounds [25]. Such a category of plant-based flavored cheeses opens interesting innovation routes for local producers and agri-food industries searching to diversify their cheese products offer [26]. 

Among plants, *Gentiana lutea* rhizomes are traditionally used in hydro-alcoholic macerations and contribute highly to bitter taste and to herbal and vegetal olfactive notes in beverages [27,28] but have never been used as a flavoring agent in cheeses. *Gentiana lutea* is a perennial plant that has naturally spread out to medium-altitude mountains (800–2500 m) all over the world [29]. Its rhizomes grow year after year by accumulating flavoring secondary metabolites such as secoiridoidal and iridoid glycosides or volatile compounds [30,31,32]. Recent studies could classify the chemical composition of *Gentiana lutea* roots in the function of geographical origin from different french mountains and of growing conditions [31,33].

The objectives of this study are: (i) to describe a novel flavored cheese using *Gentiana lutea* rhizomes and (ii) to evaluate the taste and olfactive perception of gentian flavored cheeses in regards to their chemical composition. The objective was also to discuss the infusion process in relation to the milk origin and provenance of *Gentiana lutea* during its infusion in curds. 

## 2. Materials and Methods 

### 2.1. Experimental Design

#### 2.1.1. *Gentiana Lutea* Rhizomes Sampling

*Gentiana Lutea* rhizomes were collected during the summer of 2018 in triplicates in two different sites: Chapelle des Bois in Jura (J) and Picherande in Massif Central (MC). They were harvested with the same physiological stage C [34] to take into account the variability of the site. All rhizomes were cleaned from their residual earth, manually sliced into 1–2 cm pieces, dried at 40 °C, ground to a fine powder and stored at 4 °C prior to being used as a flavoring agent in goat cheeses.

#### 2.1.2. Flavored and Unflavored Goat Cheeses Elaboration

Goat cheeses were elaborated at the cheese farm « GAEC des trèfles à quatre feuilles » (Brousse, France). Unpasteurized raw goat milk from the Alpine breed was harvested in May 2022 and poured into a thermoregulated tank at 25 °C, then acidified with rennet (0.3 g/kg) and indigenously inoculated with whey coming from the precedent goat cheese production. The clotting was left for one night, and the one-night developed curds were manually cut with a stainless-steel ladle and poured into cheese molds and left for four hours to remove the surplus whey. The corresponding molded cheeses, without gentian infusion, were named CGC (Control Goat Cheese). A total of 20 pieces of CGC of 50 g weight each were available for sensory and volatile, and non-volatile analysis.

The gentian flavored cheeses were obtained by an additional step of one-night infusion of the previously molded cheeses in a 5 g·L^−1^ *Gentiana lutea* rhizomes solution extracted in goat milk whey for one additional night. Two types of gentian flavored goat cheeses were obtained: the ones with *Gentiana lutea* rhizomes from Jura were named JGC, and those from the Massif Central were named as MCGC. A total of 20 JGC and 20 MCGC flavored goat cheeses of 50 g weight each were available for sensory and biochemical analysis.

Milk moisture, total solids, lipids and proteins were measured with a FoodScan analyzer (Foss System, Hillerød, Denmark). pH was measured with an Ingold calibrated pH meter (Ingold France, Paris, France), and Total acidity was volumetrically measured according to the Dornic method [35]. The physico-chemical composition of goat and cow milk is presented in Table 1.

### 2.2. Microbiological Analysis of Gentian Flavored and Unflavored Cheeses

25 g of flavored and unflavored goat cheese samples (25 g) were extracted in 225 mL of buffered peptone water for 2 min in a stomacher bag (BagMixer CC, Puycapel, France). Cheese total aerobic mesophilic microflora was enumerated by spread plating after serial dilution on PCA medium (CM0325, Oxoid, Thermo Fisher Diagnostics, Dardilly, France). Plates were incubated for 48 H at 30 °C [36]. Spread plating on OGA medium CM0545, Oxoid, Thermo Fisher Diagnostics, Dardilly, France) was also used to enumerate yeasts and molds after an incubation of 72 H at 25 °C [37]. Lactic acid bacteria were also enumerated by spread plating after serial dilution on MRS agar (CM0361, Oxoid, Thermo Fisher Diagnostics, Dardilly, France) medium after an incubation time of 48 H at 30 °C [38].

### 2.3. Volatile and Non-Volatile Analysis 

#### 2.3.1. Analysis of Volatile Compounds by Head-Space—Solid Phase MicroExtraction—Gas Chromatography—Mass Spectrometry (HS-SPME-GC-MS)

Volatile compounds were extracted from all the goat samples by headspace solid-phase microextraction and analyzed by GC–MS (HS-SPME-GC-MS), with a three-phase fiber (divinylbenzene (DVB)/Carboxen (CAR)/polydimethylsiloxane (PDMS), 50/30 µm, Supelco) was used. The protocol was adapted from the previous literature [27,39]. 

1 g of flavored and unflavored cheese samples and 10 mL of flavored and unflavored whey samples with a systematic addition of 100 uL of the internal standard of 100 mg·L^−1^ (2-methyl-3-heptanone) were put in a 20 mL vial. Each vial was sealed air-tight with a Teflon septum and aluminum caps and incubated in a water bath at 40 °C for 30 min. Then the fiber was exposed to the sample headspace for 30 min and was desorbed for 10 min into GC–MS. The analyses were repeated three times for each biological triplicate: CGC (Control unflavored Goat Cheese), JGC (Jura gentian flavored Goat Cheese), MCGC (Massif Central gentian flavored Goat Cheese), gathering 9 analyzed flavored and unflavored cheese samples.

The chromatographic equipment consisted of a mass spectrometer (Agilent 5975C-VLMSD, working in scan mode from *m*/*z* 29 to 400 with an electronic impact at 70 eV) paired with an Agilent 7890A gas chromatograph fitted with a split/splitless injector (240 °C). The volatile separation was carried out on a capillary column HP5 of 60 m × 0.32 mm (J&W Scientific, Folton, CA, USA), with a film thickness of 0.25 µm, using helium at 1.5 mL·min^−1^ as carrier gas. The temperature of the oven increased at a rate of 3 °C·min^−1^ from 40 °C to 240 °C and was maintained at 240 °C for 5 min. The injection was conducted in spitless mode at 240 °C and. Cheese volatile compounds were identified by matching their spectral fragmentation with pure standards and those provided by the mass spectral library of the National Institute of Standards and Technology (NIST) and the Wiley Registry (WILEY).

#### 2.3.2. Analysis of Bitter Compounds 

The unflavored and gentian flavored whey at 5 g·L^−1^ were simply filtered on 0.45 um (PTFE filters) prior to analysis. The conditions used by Ultra High-Performance Liquid Chromatography—Diode Array Detector (UHPLC-DAD) for bitter separation among gentiopicroside, loganic acid, swertiamarin and amarogentine in gentian-based matrices are identical to those previously described [33]. Yellow gentian rhizomes were extracted in a methanolic solution at 10 g·L^−1^, filtered and further analyzed. The contents of bitter compounds were expressed in g per 100 g of dried gentian.

Flavored and unflavored cheese samples were extracted at 100 g·L^−1^ in a methanolic solution during 24 H of heating at 100 °C under reflux and stirring. The supernatant was separated from the solid phase by centrifugation and filtration on 0.45 μm (PTFE filters) and poured in 2 mL UHPLC vials prior to bitter analysis. The contents of bitter compounds were expressed in g·kg^−1^ of goat cheese.

### 2.4. Sensory Analysis

Flavored and unflavored goat cheese sensory analysis took place in the sensory laboratory at VetAgro Sup (Lempdes, France), equipped with individual booths. The sensory panel consisted of 17 panelists (fourteen females and three males) who were experienced in tasting dairy products (more than 100 h on sensory characterization on cheeses).

A triangle test was carried out to evaluate the sensitivity of panelists to differentiate the bitter perception among flavored goat cheeses (JCG and MCGC), according to ISO 4120 standards. A total of 3 samples of cheese, 2 of which randomly belonged to the same treatment (i.e., JCG or MCGC), were offered for tasting (serving temperature 20 °C), and panelists were asked to identify the odd sample. Data were collected with the Tastel software (version 2019; ABT Informatique, Rouvroy-sur-Marne, France). The correct answers were counted and compared to the minimum number of correct judgments that should be obtained with a probability level of 0.05 for a statistical significance of the discriminative test [40].

The panelists were further trained to rate gentian bitter perception on goat cheeses. For that, different infusions of gentian rhizomes in goat whey at various concentrations of 0, 1 and 5 g·L^−1^ were used to calibrate the gentian bitterness rating of cheeses considering the scale of bitterness perceived from 0 (no bitterness, infusion 0 g/L) to 10 (high bitterness, infusion of 5 g/L). All Panelists tasted the three kinds of goat cheese, CGC, JGC and MCGC, and they were asked to give free descriptors based on visual, olfactive, gustative, textural and hedonic characteristics perceived during cheese tasting. A word cloud based on the number of descriptors used by the whole panel enabled a sensory snapshot of the sensory characteristics of the flavored and unflavored cheeses.

### 2.5. Statistical Analysis

Statistical data analysis was carried out using R studio software. Student’s *t*-test and one-way analysis of variance (ANOVA) were performed on the biological triplicates of microbiological and chemical variables, followed by a Tukey’s HSD (honest significant difference) post hoc test, in order to evaluate the significant differences (*p*-value < 0.05) between the three typologies of cheeses. For the values of volatile compounds relative area, we consider a statistical difference between the three typologies of cheeses, considering a *p*-value below 0.01, in order to identify the most reliable volatile compounds in cheese differentiation [41].

For sensory analysis, the Shapiro–Wilk test was first used to evaluate if the distribution of bitterness scores followed a normal law. As this condition was not checked, sensory data were analyzed using the non-parametric Friedman test followed by Nemenyi post hoc test. The free vocabulary description of each cheese was analyzed by pooling together descriptors presenting the same sensations. We consider only descriptors with a minimum of 2 citations by the whole sensory panel.

Principal component analysis using the number of citations of sensory descriptors and volatile compounds relative area for the three typologies of cheeses was carried out on OriginPro 2020 (OriginLab Corporation, Northampton, MA, USA).

## 3. Results

### 3.1. Microbiological Analysis

**Figure 1:**
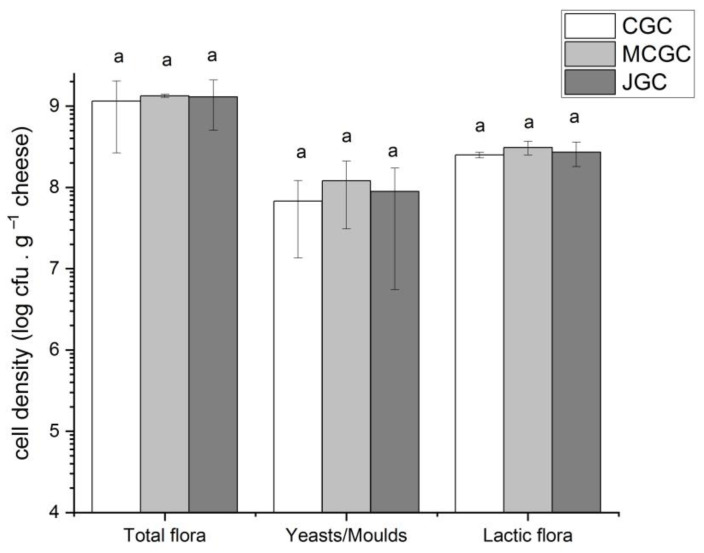
Microbiological counts (cfu/g of cheese) of different microbial groups found in goat cheeses unflavored and flavored with Gentiana lutea rhizomes from Massif Central and Jura. Error bars represent the standard deviation of the three biological triplicates. The letter a indicates there is no difference (*p* > 0.05) among cheeses per type of analyzed flora.

As shown in Figure 1, the total microbiological mesophilic aerobic count ranged from mean values of 9.0 (CGC) to 9.2 log cfu/g of cheese without any statistical difference among flavored (MCGC, JGC) and unflavored (CGC) cheeses. Lactic acid bacteria represented the dominant microorganisms found on unflavored and flavored goat cheese, respectively comprised between 8.3 and 8.5 log cfu/g of cheese, without statistical differences among the three types of goat cheeses. Yeasts and molds were less abundant compared to lactic flora, regardless of the three typologies of cheeses (between 7.8 (CGC) and 8.1 (MCGC) log cfu/g). No differences appeared again among the three types of cheeses for yeasts and molds. Similar results have already been observed on Jben and Valdeteja goat cheeses [42,43]. Montel et al. (2014) indicated maximum values reached in traditional cheeses up to 10 log cfu/g and 9 log cfu/g for mesophilic aerobic flora and lactic bacteria, respectively [11]. Our study reveals that the microbial population was not different between flavored and unflavored goat cheeses due to a very short time of contact between curds and Gentiana lutea macerated whey. Nevertheless, our study focused on unripened goat cheeses, and results could have differed upon a ripening phase or if the gentian maceration had occurred during the curdling phase [42]. 

### 3.2. Chemical Analysis of Unflavored and Flavored Goat Cheeses

#### 3.2.1. Analysis of Volatile Compounds in Unflavored and Flavored Goat Cheeses

**Figure 2:**
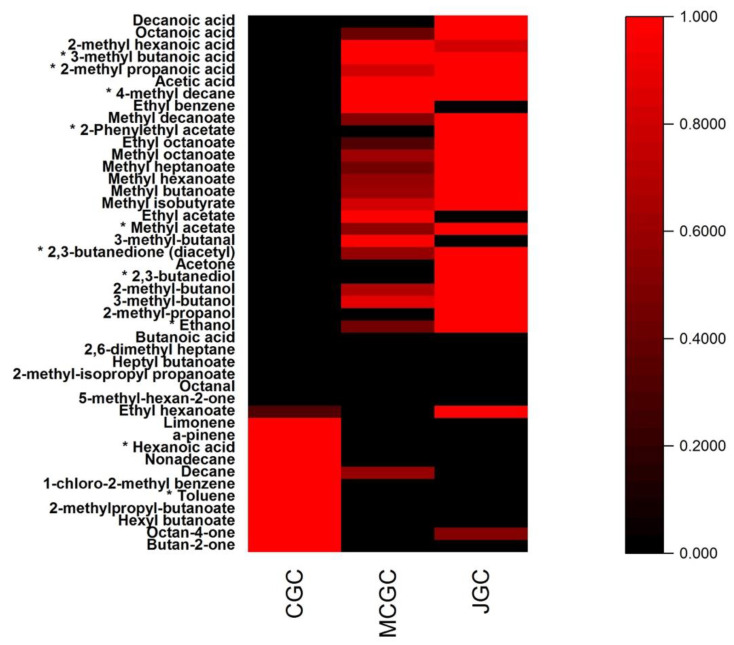
Heatmap representation of the mean values of normalized relative areas obtained for the 44 volatile compounds found in the three typologies of cheeses. * indicates that the volatile compound is a statistical biomarker (*p* < 0.01) of the gentian aromatization process in goat cheese. The color gradient scale indicates the intensity of the mean values of normalized areas found in cheeses from low (black) to high (red).

The 44 targeted volatile compounds analyzed in the three typologies of goat cheeses were represented by their normalized relative areas in the following heatmap (Figure 2). From Figure 2, it can be shown that the two typologies of cheeses (unflavored vs. flavored) were clearly distinguished by 11 volatile compounds (red color) for unflavored goat cheeses (CGC) and 27 volatile compounds for JGC and MCGC. Among those compounds, only 10 compounds (marked by an asterisk) could be statistically considered as affected by the gentian aromatization process. The mean values of the relative areas among the cheese biological replicates and the relevance of gentian aromatization biomarkers (*p* < 0.01) were presented in Table S1. The 34 other volatile compounds were common to flavored and unflavored goat cheeses. They presented no statistical differences in their relative areas among the three types of goat cheeses (Table S1). They belong to different chemical families already encountered on goat cheeses, such as ketones (butanone, octanone), aldehydes (butanal, octanal), acids (butanoic, octanoic, decanoic) [39,44,45] and other families such as alcohols, esters, alkenes, alkanes and terpenes. 

Among the 10 identified statistical biomarkers (*p* < 0.01) affected by gentian flavoring during cheesemaking, 2 volatile compounds were more present in unflavored goat cheeses compared to flavored goat cheeses: hexanoic acid and toluene. These compounds have been identified in cheeses [46,47]. Their decrease in gentian flavored goat cheeses could be putatively attributed to microbial metabolism or biotransformation (oxidation or enzymatic reactions) or due to easy leachability from goat cheeses compounds during the gentian flavoring step. Concerning the eight other compounds: ethanol, 2,3-butane-diol, diacetyl, methyl acetate, 2-phenylethyl acetate, 4-methyl decane, 2-methyl propanoic acid, and 3-methyl butanoic acid, they appeared to be enhanced on gentian flavored goat cheeses, regardless of the origin of *Gentiana lutea* rhizomes, except for 2-phenylethyl acetate and 4-methyl decane. All of these eight compounds have already been identified in goat cheeses, except 4-methyl decane, which originated from gentian rhizomes [27,31]. 4-methyl decane appeared to be more present in MGCG compared to JGC. On the opposite, 2-phenylethyl acetate was more present in JGC compared to MCGC. These two potential odor-active markers of gentian origin offer an interesting route for possible geographical distinction among gentian enriched goat cheeses.

#### 3.2.2. Analysis of Bitter Compounds Transfer from *Gentiana lutea* Rhizomes in Gentian Flavored Goat Cheeses

Table 2 indicates the mean values of the contents of seco-iridoids compounds in Gentiana lutea rhizomes, in flavored goat whey after the gentian infusion, and in flavored goat cheeses after the flavoring process. As previously found in literature, gentiopicroside and loganic acid are the two most abundant compounds present in *Gentiana lutea* rhizomes, respectively, with mean values comprised between 5.90 and 6.50% and between 1.20 and 1.55% with differences already observed among these bitter compounds in the function of harvesting origin and growing conditions [33,48]. Swertiamarin and amarogentin presented lower mean values of 0.40% and 0.02% for each geographical site. The infusion process in goat whey led to a complete recovery of loganic acid and swertiamarin extracted from the initial gentian rhizomes used for the infusion since similar values of mass percentages were observed. For gentiopicroside and amarogentin, no recovery in macerated whey was observed, except for gentiopicroside with the Jura gentian root with a mean value of mass percentage of 1%. Concerning the flavored goat cheeses, the only compound that was partially recovered during gentian rhizomes infusion was loganic acid, with concentrations ranging from 0.11 g/kg (JGC) to 0.14 g/kg (MCGC). No statistical difference was found for loganic acid content among the two flavored kinds of cheese. It was confirmed this compound was totally absent from CGC (no signal in the chromatographic elution). To our knowledge, the extraction of gentian rhizomes has already been discussed in the previous literature [27,49] with alcoholic and aqueous solvents but never on lipophilic solvents as those encountered in goat whey used for the proposed infusion protocol. When calculating the transfer ratio of loganic acid from flavored goat whey to flavored goat cheese, values from 10 for MCGG and 11 for JGC were obtained and indicated a very close diffusion step occurring during the flavoring step. Such results were in adequation with similar experiments used in cheesemaking as the curd immersion in modified milk wheys with different amounts of salts and minerals [50]. 

### 3.3. Sensory Analysis of Flavored Goat Cheese

**Figure 3:**
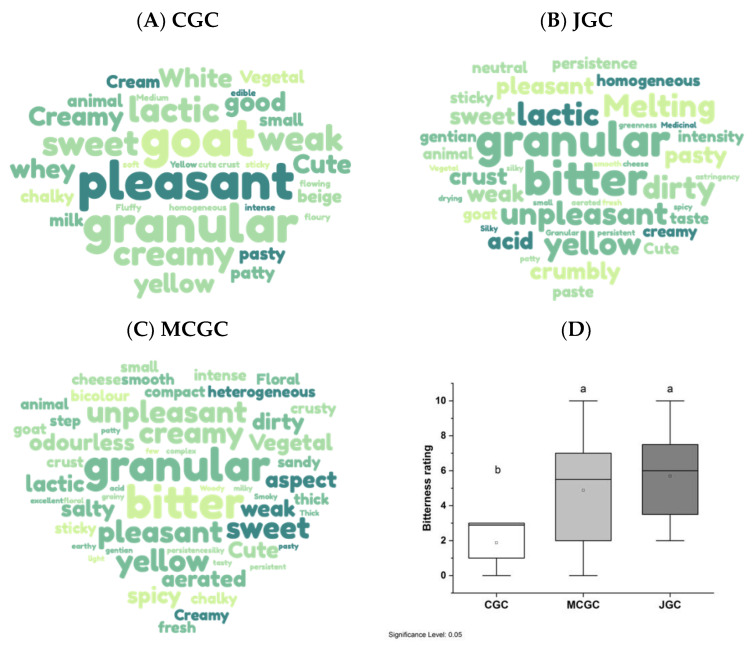
Free descriptors associated with unflavored goat cheese (**A**) and flavored goat cheeses elaborated with *Gentiana lutea* rhizomes originating from Massif Central (**B**) and Jura (**C**). The bigger the word, the more the descriptor was cited by cheese tasters, between 2 times and 16 times among the whole panel. Histogram of bitterness rating of flavored and unflavored goat cheese evaluated by the panelists (n = 17) (**D**). Letters indicate the significant difference based on mean comparison with the Friedman test applied for each tasted cheese (alpha = 5%).

Figure 3A–C presents a list of descriptors generated by the free descriptors used by the panelist to describe the flavored and unflavored goat cheeses. Interestingly the terms associated with visual, olfactive, gustative, textural and hedonic aspects were raised by the panelists during cheese tasting. The number of words associated with descriptors of flavored goat cheeses regardless of the origin of gentian (JGC and MCGC) was higher (between 105 to 110 words) compared to unflavored goat cheese (CGC). This reveals the more complex sensations perceived when tasting gentian-flavored cheese. Among these descriptors, some were common to unflavored and flavored goat cheese, such as “goat”, “animal”, “granular”, “lactic”, “creamy”, and “vegetal” with their frequencies of citations by the whole panel differing for each type of goat cheeses. Other descriptors appear unique to gentian flavored goat cheese, such as “bitter”, “spicy”, “floral”, “smoky”, and “medicinal”. A correspondence analysis carried out on the citation occurrence by panelists highlighted these differences among the three typologies of cheeses (Figure S1). Figure 3D indicates the clear difference in bitterness perception among flavored and unflavored goat cheeses, meanly rated at 1.9 for CGC and up to 4.9 and 5.7 for MCGC and JGC, respectively. The higher bitterness perceived by panelists for gentian flavored goat cheeses compared to unflavored goat cheeses presented no statistical difference in the function of the geographical origin (Jura vs. Massif Central). Such a result was corroborated by a triangle test undertaken on the flavored cheeses (JGC and MCGC). Only 7 of 17 panelists were able to differentiate the two typologies of gentian flavored cheeses, which was not statistically different (*p* > 0.05) [40].

## 4. Discussion of Sensory and Chemically Traits of Gentian Flavored and Unflavored Goat Cheeses

**Figure 4:**
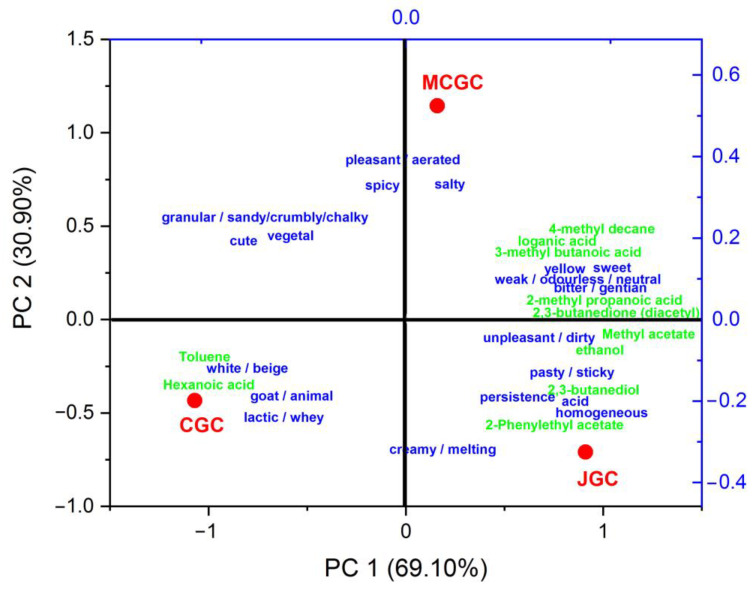
Biplot representation of the principal component analysis of the non-flavored GCG and flavored goat cheeses JGC and MCGC (red legend) based on their sensory and chemical characteristics. Loadings of the PCA model represent the number of citations of free descriptors (blue legend) associated with unflavored and gentian flavored goat cheeses (citations cited at least twice) and the relative contents of the ten volatile compounds (green legend) statistically differentiated among the three typologies of goat cheeses.

In order to evaluate the sensory and chemical characteristics of the three typologies of cheeses, a principal component analysis (Figure 4) was carried taking as loadings the number of citations (with a minimum of twice cited by the whole panelists) of free descriptors associated to unflavored and gentian flavored goat cheeses and the relative contents of the ten volatile compounds statistically differentiated among the three typologies of goat cheeses (Table S1), and the loganic acid measured in flavored cheeses (Table 2). Results of the PCA model led to a clear statistical differentiation among the cheeses along axis PC1 and PC2, explaining 69.1% and 30.9% of the total variance, respectively.

The unflavored goat cheese was characterized by a relatively higher number of citations related to color, odor and taste “white/beige”, “goat/animal”, and “lactic/whey”. They presented higher relative amounts of toluene and hexanoic acid that could confer to goat cheeses its higher perception of goaty notes compared to gentian flavored goat cheeses. Such direct and retro-olfactive notes could also be conferred by other volatile compounds, such as butyric, octanoic acid or decanoic acid [45,51], that were not necessarily statistically differentiated among the three typologies of cheeses.

Some unpleasant olfactive notes perceived in gentian flavored goat cheeses could be attributed to stinky smells from 3-methyl butanoic acid or 2-methyl propanoic acid and 4-methyl decane that were relatively more abundant in flavored goat cheeses compared to unflavored cheeses. On the opposite, pleasant notes were attributed to gentian flavored goat cheeses such as “creamy”, “sweet”, “floral”, “spicy” and “vegetal” and could reliably be associated with volatile compounds found in this study, such as 2,3-butanediol, diacetyl, methyl acetate, 2-phenylethyl acetate. Those could have been brought during the gentian infusion protocol during cheesemaking, but the microbial metabolism modification by the input of gentian chemicals could not be excluded. The relatively higher amount of ethanol in gentian flavored goat cheeses could be in relation to their “creamy/melting” characteristics due to their solvent properties that could increase the solubility of casein structures in cheeses [52]. Finally, the “bitter/gentian” taste perceived by panelists in all the gentian flavored goat cheeses (MCGC and JGC) could be attributed to the presence of loganic acid in those cheeses. Such compounds have already been identified in gentian aromatized food products such as liqueurs or flavored wines [27,53].

## 5. Conclusions

In this study, we develop flavored goat cheeses with *Gentiana lutea* rhizomes infusion in goat’s milk whey. Two distinct geographical origins of gentian rhizomes were used for cheese aromatization in comparison with unflavored goat cheeses. Microbiological analysis revealed no differences in unaged flavored and unflavored goat cheeses where lactic flora predominated the fermentative steps of cheesemaking. Gentian flavored cheeses presented an intense bitterness perception with no statistical difference in the geographical origin of gentian rhizomes. Gentian flavored goat cheeses were also characterized by less goaty and lactic/whey notes but with more herbal, vegetal, floral, sweet, spicy and creamy notes compared to unflavored goat cheeses. Such cheese sensory descriptions were discussed in the function of their relative volatile composition. Interestingly volatile compounds belonging to acids, alcohols, alkenes, ketones, and esters enabled to specify the typicity of gentian flavored goat cheeses without statistical difference in the geographical origin of gentian rhizomes, except for phenylethyl acetate that was relatively more present in flavored cheeses made with yellow gentian coming from Jura compared to Massif Central. The proposed protocol of yellow gentian flavoring used for cheesemaking could be interesting to be further developed in order to offer new sensory sensations to consumers and to give the opportunity to cheesemakers to develop innovative dairy products.

## Figures and Tables

**Table 1:** Physico-chemical characteristics of raw goat milk used in this study, in comparison with raw cow milk.

	Moisture (%)	Total Solids (%)	Lipids (%)	Proteins (%)	pH	Titrable Acidity (g/L eq Lactic Acid)
Goat milk	80.3	12.2	3.9	3.4	6.5	1.58
Cow milk	85.3	14.7	4.7	4.4	6.7	1.45

**Table 2:** Mean values and standard deviations of contents of seco-iridoids expressed in g/100 g of dried gentian rhizomes for *Gentiana lutea* rhizomes and gentian flavored goat whey and in g/kg for gentian flavored goat cheeses. ND means the compound is no more detected under our chromatographic conditions. Values with a common superscript are not significantly different (*p* < 0.05).

	*Gentiana lutea* Rhizomes (g/100 g Dried Rhizomes)		*Gentiana lutea* Flavored Goat Whey (g/100 g Dried Rhizomes)		*Gentiana lutea* Flavored Goat Cheese (g/kg Cheese)	
	Massif Central	Jura	Massif Central	Jura	MCGC	JGC
Gentiopicroside	6.50 ± 0.22 ^(a)^	5.90 ± 0.18 ^(b)^	ND	1.00 ± 0.18	ND	ND
Loganic acid	1.55 ± 0.10 ^(a)^	1.20 ± 0.09 ^(b)^	1.49 ± 0.20 ^(a)^	1.26 ± 0.19 ^(a)^	0.14 ± 0.02 ^(a)^	0.11 ± 0.01 ^(a)^
Swertiamarin	0.40 ± 0.05 ^(a)^	0.40 ± 0.04 ^(a)^	0.47 ± 0.10 ^(a)^	0.43 ± 0.09 ^(a)^	ND	ND
Amarogentin	0.02 ± 0.01 ^(a)^	0.02 ± 0.01 ^(a)^	ND	ND	ND	ND

## Data Availability

The data are available from the corresponding author.

## Supplementary Materials

The following supporting information can be downloaded at: https://www.mdpi.com/article/10.3390/foods12030468/s1, Figure S1: Correspondence map on the contingency table containing the frequency of the sensory attributes (triangle red labels) cited by the panelists. The blue circles represent the three typologies of goat cheeses in the correspondence analysis.; Table S1: Mean values of relative area, normalized to internal standard, analyzed on three biological triplicates for each category of goat cheeses. The letters indicate the significant difference based on mean comparison with the Tuckey method ap-plied for each volatile compound (*p*-value < 0.01). The bold lines correspond to volatile compounds that present statistical differences among the three typologies of goat cheeses.
