# Supplementary material for: Development of a Novel Flavored Goat Cheese with Gentiana lutea Rhizomes
Source: Foods. 2023 Jan 19;12(3):468. doi: 10.3390/foods12030468 (PMC9914017; doi:10.3390/foods12030468)

foods

MDPI

Supplementary Materials:

Table S1. Mean values of relative area, normalized to internal standard, analyzed on three biological triplicates for each category of goat cheeses. The letters indicate the significant difference based on mean comparison with the Tuckey method applied for each volatile compound (p-value < 0.01). The bold lines correspond to volatile compounds that present statistical differences among the three typologies of goat cheeses.

Family	Compound	CGC	MCGC	JGC
	**Ethanol**	**0.043 (a)**	**0.056 (ab)**	**0.071 (b)**
	2-methyl-propanol	0.003 (a)	0.003 (a)	0.004 (a)
Alcohols	3-methyl-butanol	0.020 (a)	0.028 (a)	0.029 (a)
	2-methyl-butanol	0.007 (a)	0.009 (a)	0.010 (a)
	**2,3-butanediol**	**0.001 (a)**	**0.001 (ab)**	**0.003 (b)**
	Acetone	0.017 (a)	0.017 (a)	0.020 (a)
	Butan-2-one	0.003 (a)	0.002 (a)	0.002 (a)
Ketones	**2,3-butanedione (diacetyl)**	**0.001 (a)**	**0.004 (b)**	**0.006 (b)**
	Octan-4-one	0.007 (a)	0.005 (a)	0.006 (a)
	5-methyl-hexan-2-one	0.001 (a)	0.001 (a)	0.001 (a)
Aldehydes	Octanal	0.001 (a)	0.001 (a)	0.001 (a)
	3-methyl-butanal	0.002 (a)	0.003 (a)	0.002 (a)
	**Methyl acetate**	**0.004 (a)**	**0.053 (ab)**	**0.091 (b)**
	Ethyl acetate	< 0.001 (a)	0.003 (a)	< 0.001 (a)
	Methyl isobutyrate	0.002 (a)	0.006 (a)	0.007 (a)
	Methyl butanoate	0.011 (a)	0.068 (a)	0.101 (a)
	2-methyl-isopropyl propanoate	0.001 (a)	0.001 (a)	0.001 (a)
	Methyl hexanoate	0.029 (a)	0.351 (a)	0.590 (a)
	Hexyl butanoate	0.002 (a)	0.001 (a)	0.001 (a)
Esters	2-methylpropyl-butanoate	0.002 (a)	0.001 (a)	0.001 (a)
	Heptyl butanoate	0.001 (a)	0.001 (a)	0.001 (a)
	Ethyl hexanoate	0.009 (a)	0.008 (a)	0.011 (a)
	Methyl heptanoate	< 0.001 (a)	0.006 (a)	0.012 (a)
	Methyl octanoate	0.017 (a)	0.636 (a)	1.033 (a)
	Ethyl octanoate	0.002 (a)	0.003 (a)	0.005 (a)
	**2-Phenylethyl acetate**	**0.004 (a)**	**0.004 (a)**	**0.007 (b)**
	Methyl decanoate	0.006 (a)	0.241 (a)	0.467 (a)
Alkenes	**Toluene**	**0.002 (a)**	**0.001 (b)**	**0.001 (b)**
	Ethyl benzene	< 0.001 (a)	0.003 (a)	< 0.001 (a)
	1-chloro-2-methyl benzene	0.002 (a)	< 0.001 (a)	< 0.001 (a)
Alkanes	Decane	0.021 (a)	0.018 (a)	0.014 (a)
	**4-methyl decane**	**ND**	**0.001 (b)**	**< 0.001 (b)**
	2,6-dimethyl heptane	0.001 (a)	0.001 (a)	0.001 (a)
	Nonadecane	0.004 (a)	0.003 (a)	0.003 (a)
Acids	Acetic acid	0.001 (a)	0.004 (a)	0.004 (a)
	**2-methyl propanoic acid**	**0.001 (a)**	**0.005 (b)**	**0.006 (b)**
	Butanoic acid	0.006 (a)	0.006 (a)	0.006 (a)
	**3-methyl butanoic acid**	**0.005 (a)**	**0.023 (b)**	**0.023 (b)**
	2-methyl hexanoic acid	0.002 (a)	0.008 (a)	0.007 (a)
	**Hexanoic acid**	**0.020**	**ND**	**ND**
	Octanoic acid	0.002 (a)	0.019 (a)	0.044 (a)
	Decanoic acid	< 0.001 (a)	< 0.001 (a)	0.010 (a)
Terpenes	α-pinene	0.002 (a)	< 0.001 (a)	0.001 (a)
	Limonene	0.010 (a)	0.005 (a)	0.005 (a)

Figure S1 : Correspondence map on the contingency table containing the frequency of the sensory attributes (triangle red labels) cited by the panelists. The blue circles represent the three typologies of goat cheeses in the correspondence analysis.

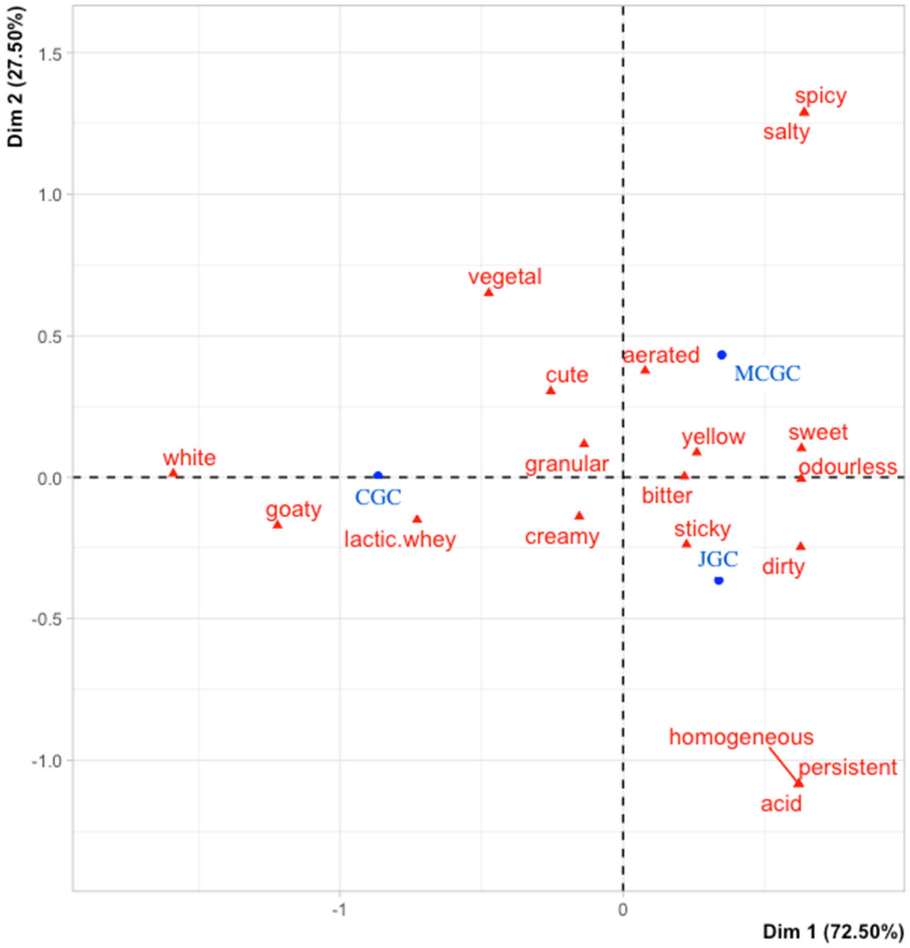

Supplement: Supplementary file 1 [file foods-12-00468-s001.zip › foods-2143533-supplementary.pdf]
